# Reprogramming of the chick retinal pigmented epithelium after retinal injury

**DOI:** 10.1186/1741-7007-12-28

**Published:** 2014-04-17

**Authors:** Agustin Luz-Madrigal, Erika Grajales-Esquivel, Alexander McCorkle, Ashley M DiLorenzo, Karla Barbosa-Sabanero, Panagiotis A Tsonis, Katia Del Rio-Tsonis

**Affiliations:** 1 Department of Biology, Miami University and Center for Visual Sciences at Miami University (CVSMU), Oxford, OH 45056, USA; 2 Department of Biology, University of Dayton and Center for Tissue Regeneration and Engineering at the University of Dayton (TREND), Dayton, OH 45469, USA

**Keywords:** Regeneration, Retina, Transdifferentiation

## Abstract

**Background:**

One of the promises in regenerative medicine is to regenerate or replace damaged tissues. The embryonic chick can regenerate its retina by transdifferentiation of the retinal pigmented epithelium (RPE) and by activation of stem/progenitor cells present in the ciliary margin. These two ways of regeneration occur concomitantly when an external source of fibroblast growth factor 2 (FGF2) is present after injury (retinectomy). During the process of transdifferentiation, the RPE loses its pigmentation and is reprogrammed to become neuroepithelium, which differentiates to reconstitute the different cell types of the neural retina. Somatic mammalian cells can be reprogrammed to become induced pluripotent stem cells by ectopic expression of pluripotency-inducing factors such as Oct4, Sox2, Klf4, c-Myc and in some cases Nanog and Lin-28. However, there is limited information concerning the expression of these factors during natural regenerative processes. Organisms that are able to regenerate their organs could share similar mechanisms and factors with the reprogramming process of somatic cells. Herein, we investigate the expression of pluripotency-inducing factors in the RPE after retinectomy (injury) and during transdifferentiation in the presence of FGF2.

**Results:**

We present evidence that upon injury, the quiescent (p27^Kip1^+/BrdU-) RPE cells transiently dedifferentiate and express *sox2*, *c-myc* and *klf4* along with eye field transcriptional factors and display a differential up-regulation of alternative splice variants of *pax6*. However, this transient process of dedifferentiation is not sustained unless FGF2 is present. We have identified *lin-28* as a downstream target of FGF2 during the process of retina regeneration. Moreover, we show that overexpression of *lin-28* after retinectomy was sufficient to induce transdifferentiation of the RPE in the absence of FGF2.

**Conclusion:**

These findings delineate in detail the molecular changes that take place in the RPE during the process of transdifferentiation in the embryonic chick, and specifically identify Lin-28 as an important factor in this process. We propose a novel model in which injury signals initiate RPE dedifferentiation, while FGF2 up-regulates Lin-28, allowing for RPE transdifferentiation to proceed.

## Background

Several vertebrate species have the capacity to transdifferentiate the retinal pigmented epithelium (RPE) to retina (for reviews, see [1,2]). In the chick, the process of RPE transdifferentiation was first described based on histological observations [3]. We previously demonstrated that after retina removal from chick eyes at embryonic day (E) 4 to 4.5 (Stage 24 to 25, according to Hamburger and Hamilton Stages [4]) and in the presence of fibroblast growth factor 2 (FGF2), the RPE loses its pigmentation and transdifferentiates to become a neuroepithelium, co-expressing the retinal progenitor markers Pax6 and Chx10 through FGF/FGF-Receptor (FGFR)/Mitogen-activated protein kinase (MAPK) and extracellular-signal-regulated kinase (ERK) signaling cascade [5,6]. Concomitantly with RPE transdifferentiation, the transcriptional factor Mitf, an RPE-specific marker, is down-regulated, suggesting a change in cell fate of the injured RPE. The ectopic expression of Mitf is sufficient to inhibit RPE transdifferentiation, likely inhibiting the up-regulation of *pax6* expression [6]. During retina regeneration from the RPE, the newly generated neuroepithelium eventually differentiates into all major cell types found in the retina, and the differentiation pattern follows the same order as it does during normal development [5]. The ability of RPE cells to transdifferentiate ceases as embryonic development proceeds beyond E4.5 [3]. However, the ectopic expression of *pax6* is sufficient to induce RPE transdifferentiation in the intact developing chick eye up to E14 (Stage 35) [7]. In chick RPE cultures, overexpression of different pro-neural transcriptional factors such as *sox2*, *ash1*, *ath5*, *neuroD*, *neurogenin1*, *neurogenin3*, *cath5* and *msx2* can promote the transdifferentiation of the RPE into neuronal cells (reviewed in [1]). By contrast, there are several factors associated with RPE specification, including Mitf*,* Otx2*,* Wnt13*,* BMP*,* Shh and Activin [6,8-15]. The inactivation of Wnt/beta-catenin signaling in the embryonic mouse RPE results in down-regulation of RPE-specific factors Mitf and Otx2 and expression of neural retina markers Chx10 and Rx [9,10].

Recently, it has been demonstrated that somatic mammalian cells can be reprogrammed to become induced pluripotent stem cells by ectopic expression of pluripotency-inducing factors Oct4, Sox2, c-Myc and Klf4 as well as by the combination of Oct4, Sox2, Nanog and the RNA-binding protein Lin-28 [16,17]. Among all these transcriptional factors, Oct4 (Pou5f1), Nanog and Sox2 are key factors that maintain embryonic stem cell identity [18]. More recently, efficient differentiation of induced pluripotent stem cells into neural retina cells has been demonstrated, suggesting the possibility of using these cells for clinical therapies [19]. Other studies have used a specific set of factors to convert fibroblasts directly into induced neural cells [20]. For example, mouse fibroblasts can be directly converted into induced neural cells by overexpressing Ascl1, Brn2 and Myt1l. However, these induced cells lack the potential to generate diverse neural subtypes [21]. In another work, transient expression of Oct4, Sox2, c-Myc and Klf4 was sufficient to induce transdifferentiation of mouse fibroblasts to neural stem/progenitors cells [22] that can be expanded and differentiate in multiple neuronal subtypes and glial cells. Although all these methods of reprogramming yield cells with similar characteristics to the target cells, it is still unknown if these reprogrammed cells are able to recapitulate the natural process of differentiation or whether the induced pluripotent stem cells or induced neural cells retain the epigenetic memory of their origin. Importantly, aberrant expression of pluripotency genes, incomplete demethylation of specific promoters, viral integration and, more prominently, cancer [23-26] have been reported as a result of reprogramming. Moreover, from the medical point of view, the possibility to integrate these cells into somatic tissue remains unclear.

As an alternative, the study of transdifferentiation and regeneration could provide important information regarding maintenance of pluripotency, dedifferentiation processes, factors involved in cell reprogramming and integration of the cells in the regenerated tissue [27]. Initial studies have shown that among the pluripotency-inducing factors, *sox2*, *c-myc* and *klf4* are the common factors expressed during lens and limb regeneration in newts and during fin regeneration and Müller glia dedifferentiation in zebrafish [28-30]. More recently, it was demonstrated that in mammals Lin-28 can enhance tissue repair in several contexts including improved hair regrowth and accelerated regrowth of cartilage, bone and mesenchyme after ear and digit injuries [31].

Lin-28 is an important regulator of *let-7* miRNAs, and it has a functional role in organismal growth and metabolism, tissue development, somatic reprogramming and cancer (reviewed in [32]). During *in vitro* differentiation of mouse embryonic carcinoma cells to neural and glial fates, Lin-28 can alter the cell fate independently of *let-7*; in addition, overexpression of Lin-28 increases neurogenesis in the same cell types [33]. *In vitro* and *in vivo* experiments have demonstrated that Lin-28 regulates the translation and stability of a large number of mRNAs including cell cycle regulators, splicing factors, metabolic enzymes and RNA-binding proteins [31,34-38]. All this evidence strongly suggests that Lin-28 can have a pivotal role in tissue regeneration.

Consistent with this idea, we analyzed the expression of pluripotency-inducing factors, including Lin-28, during RPE transdifferentiation, using the embryonic chick model of retina regeneration. Among all the factors, *sox2*, *c-myc* and *klf4* were transiently up-regulated in the injured RPE (after retinectomy) along with eye field transcriptional factors, achaete-scute complex homolog 1 (*ascl1* also known as chicken achaete-scute homolog (CASH-1)) and differential up-regulation of alterative splice variants of *pax6*. By contrast, *lin-28* was significantly up-regulated only in the presence of FGF2 in retinectomized eyes. Moreover, Lin-28 overexpression in the injured RPE was sufficient to induce RPE transdifferentiation.

These results establish a two-step dedifferentiation process. First, upon injury there is an activation of gene expression for *sox2*, *c-myc* and *klf4*, concomitantly with the up-regulation of eye field transcriptional factors. Second, in the presence of FGF2, *lin-28* is up-regulated, suggesting a correlation between the expression of *lin-28* and the process of transdifferentiation. Overexpression of Lin-28 in the injured RPE was sufficient to induce RPE transdifferentiation in the absence of an external source of growth factors. To our knowledge, these data provide the first evidence that Lin-28 plays an important role in retina regeneration via RPE transdifferentiation. This is also the first detailed study on the molecular profile of the RPE during the process of dedifferentiation.

## Results and discussion

### Expression of pluripotency-inducing factors in the ciliary margin and retinal pigmented epithelium

**Figure 1:**
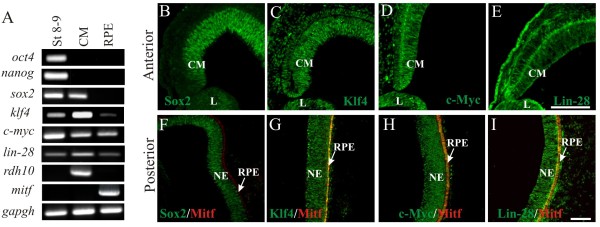
**Expression of pluripotency-inducing factors in the developing chick eye. (A)** RT-PCR analysis of pluripotency-inducing factors at Stage 8 to 9 and ciliary margin (CM) and retinal pigmented epithelium (RPE) at Stage 24 (E4). Expression of the housekeeping gene *gapdh* was used as an internal control. *rdh10* and *mitf* are specifically expressed in the CM and RPE respectively and were used as controls for the specificity of the tissues. Immunohistochemical staining using antibodies against **(B)** Sox2, **(C)** Klf4, **(D)** c-Myc and **(E)** Lin-28 in the anterior region of E4 eyes (in green). In the posterior region, **(F)** Sox2, **(G)** Klf4, **(H)** c-Myc and **(I)** Lin-28 were tested along with Mitf (microphthalmia-associated transcriptional factor; in red). The scale bar in panel **E** represents 100 μm and also applies to panels **B**, **C** and **D**. Scale bar in panel **I** represents 50 μm and applies to panels **F**, **G** and **H**. CM, ciliary margin; L: lens; NE: neuroepithelium; RPE, retinal pigmented epithelium.

To dissect the possible role of pluripotency-inducing factors during RPE transdifferentiation, we decided first to analyze the expression of these factors in E4 eyes (Stage 24, when retinectomies are performed). The presence of transcripts was analyzed by reverse transcriptase-polymerase chain reaction (RT-PCR) from RNA collected from the ciliary margin or RPE. In order to keep the integrity and specificity of all the tissues collected, we used laser capture microdissection. We found that *sox2*, *c-myc*, *klf4* and *lin-28* mRNAs were expressed in the ciliary margin, while only *klf4*, *c-myc* and *lin-28* were detected in the RPE (Figure 1A). Consistent with the RT-PCR analysis, immunofluorescence staining demonstrated that Sox2, c-Myc, Klf4 and Lin-28 were present in both the ciliary margin and neuroepithelium (Figure 1B-I and Additional file 1: Figure S1A-D). By contrast, only Klf4*,* c-Myc and Lin-28 were present in the RPE at E4 and E7 (Figure 1G-I and Additional file 1: Figure S1A,C,D). In agreement with our results, Sox2 has been reported to be expressed in the presumptive neural retina and is down-regulated in the presumptive RPE at E4 to 4.5 (Stage 24 to 25) [39].

Among these factors, it has been reported that Klf4 plays a critical role in neurogenesis and neural migration during cerebral cortex development in mouse [40]. It could be possible that Klf4 has a similar role in retina development. In chick embryos, *klf4* mRNA was detected in the neural folds and in the neural tube at Stages 8 to 9. Later, at Stage 27, *klf4* was detected in the face and neck region [41]. In our experiments, we detected Klf4 ubiquitously in the ciliary margin, neuroepithelium and RPE (Figure 1A,C,G and Additional file 1: Figure S1C).

In chicken embryos, Lin-28 is ubiquitously expressed in the presumptive limb primordium at Stages 15 to 16, and in other tissues including the neuroepithelium of the optic cup and in the otic vesicle at E2.5 to 3 (Stages 17 to 18) [42]. In mouse, Lin-28 is present in the retina at embryonic days E8.5 to E17.5 [42,43]. We detected Lin-28 in the chick ciliary margin, RPE and neuroepithelium at E4 (Stage 24) (Figure 1A,E,I) and at E7 (Additional file 1: Figure S1D), suggesting that the presence of this protein could be necessary for growth and differentiation of these tissues.

Among all the factors controlling the regulatory network in embryonic stem cells, Oct4 and Nanog are considered the ‘key partner core’ of transcriptional regulators (for review see [44,45]). Expression of the avian homologs of *oct4* (*cPouV*) and *nanog* was demonstrated early in the developing chick at Stages 8 to 9 [46]. We were unable to detect mRNAs of *oct4* and *nanog* in the ciliary margin or RPE at the embryonic days analyzed, but we did confirm their expression in chick embryos at Stages 8 to 9 (Figure 1A). Although we found the expression of *sox2*, *c-myc* and *klf4* in the ciliary margin, the absence of *oct4* and *nanog* points out that these cells are not pluripotent but may retain some properties of stem cells. Despite the fact that Sox2, c-Myc, Klf4 and Lin-28 are considered pluripotency-inducing factors when used for reprograming in combination with Oct4 and Nanog, these factors have other functions during eye and retina development [39,47-50].

### In the injured eye, the retinal pigmented epithelium dedifferentiates before entering the cell cycle and expresses *sox2*, *c-myc* and *klf4*

It is known from several organisms, that transdifferentiation occurs by the following steps: transient dedifferentiation, proliferation and differentiation into the new linage [1,51]. However, the time of dedifferentiation and proliferation is highly dependent on the type of damage and model of regeneration. For example, in zebrafish retina, different damage paradigms including light lesions (acute and chronic), chemical treatments that kill retina neurons (ouabain) and mechanical insults to the retina (needle stabbing) ultimately result in regeneration of the lost neurons by Müller glia transdifferentiation; however, the time at which Müller glia dedifferentiate or enter the cell cycle varies between the treatments. Dedifferentiation events have been reported as early as 4 h for the acute light lesion model, about 15 h for the stabbing model and up to 31 h for chronic light lesion cases. Signs of cells entering the cell cycle have been observed 24 to 72 h post lesion (for reviews see [1,52,53]. Interestingly, retina damage caused by different concentrations of ouabain generates extensive cell death of retina neurons and promotes the surviving Müller glia to proliferate robustly within a 3- to 12-day period [54]. During lens regeneration in the newt, the pigmented epithelial cells from the dorsal iris that are responsible for replacing the missing lens express nucleostemin (a stem cell marker) 2 days after lentectomy. This is followed by the loss of pigmentation and cell identity, facilitating the subsequent proliferation that takes place 4 days later [55]. Notably, inhibiting the cell cycle using a Cdk2 inhibitor does not block the process of dedifferentiation [56].

To better understand and characterize the process of RPE transdifferentiation, we analyzed the proliferative status of the RPE. We performed complete retinectomies in E4 (Stage 24) chick eyes in the presence or absence of FGF2, and the embryos were collected at 6 and 24 hours post-retinectomy (h PR) to examine 5-bromo-2′-deoxyuridine (BrdU) incorporation and the cyclin-dependent kinase inhibitor 1B (p27^Kip1^) for cell cycle arrest. At 6 h PR, in the absence (retinectomy only) or presence of FGF2, we did not observe BrdU-positive cells in the posterior RPE (Additional file 2: Figure S2A,E). By contrast, a large proportion of p27^Kip1^-positive cells were found in the posterior RPE at 6 h PR regardless of the presence of FGF2 (Additional file 2: Figure S2B,F), suggesting that at this time point the cells were still arrested in the cell cycle and as a consequence proliferation was blocked. However, BrdU-positive cells were detected in the RPE at 24 h PR only in the presence of FGF2 (Additional file 2: Figure S2G), when the RPE became p27^Kip1^-negative (Additional file 2: Figure S2H), suggesting that RPE cells had entered the cell cycle. We did not observed BrdU-positive cells in the RPE at 24 h PR in the absence of FGF2 (Additional file 2: Figure S2C) when the RPE was still p27^Kip1^-positive (Additional file 2: Figure S2D); therefore, FGF2 is necessary for the cell cycle entry, and eventually for RPE transdifferentiation [6].

**Figure 2:**
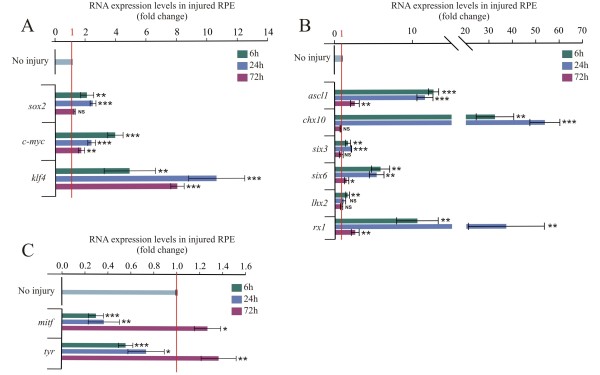
**Pluripotency inducing factors *sox2*, *c-myc *and *klf4*, and eye field transcriptional factor expression is increased in the retinal pigmented epithelium after retina removal.** Quantitative RT-PCR analysis at 6, 24 and 72 hours post-retinectomy (injury) of **(A)***sox2*, *c-myc* and *klf4*; **(B)** eye field transcriptional factors *six3*, *six6*, *lhx2* and *rx1* and the progenitor markers *ascl1* and *chx10*; and **(C)** Retinal pigmented epithelium (RPE)-specific factors *mitf* and *tyr*. The expression levels were normalized with intact RPE (no injury). The analysis was performed using three independent biological samples (*n* = 3) in triplicate and the comparative cycle threshold (2^-ΔΔCt^) method was used to determine relative changes in transcripts compared with *gapdh* mRNA levels. Significance was determined with unpaired Student’s t-test by comparing each time point with the intact RPE (no injury). Error bars represent standard error of the mean. *P < 0.05; **P < 0.01; ***P < 0.001, compared with intact RPE. NS, non-significant; RPE, retinal pigmented epithelium.

To analyze the process of dedifferentiation, we wondered if injury (retinectomy) was sufficient to initiate changes in gene expression of pluripotency-inducing factors, genes associated with the RPE specification, and genes associated with retina progenitors, as well as eye field transcriptional factors. We used the following time points of analysis as a reference, 6 h PR (when the RPE cells were still arrested in the cell cycle, even in the presence of FGF2), 24 h PR (when the RPE cells entered the cell cycle in the presence of FGF2) and 72 h PR (when the RPE transdifferentiates in the presence of FGF2 [5,6]). mRNA levels for all different genes were evaluated by quantitative RT-PCR (RT-qPCR) using RPE samples collected by laser capture microdissection. Surprisingly, at 6 h PR, we observed activation of gene expression of *sox2*, *c-myc* and *klf4* and over the basal levels detected in uninjured eyes. However, the expression of *sox2* decreased by 72 h PR to the basal levels (*P* = 0.072, *n* = 3) (Figure 2A). Although the injury was sufficient to up-regulate *sox2*, *c-myc* and *klf4* (Figure 2A), which are present in retina progenitors (Figure 1B-D), the absence of the transcripts for *oct4* and *nanog* that are present in embryonic stem cells suggest that the RPE cells do not become pluripotent, but do acquire some plasticity. In agreement with our results, *in vitro* culture of RPE cells, isolated from adult human donor eyes, showed high levels of *c-myc* and *klf4* compared to human embryonic stem cells, however, *oct4* and *nanog* were not detected by immunostaining or RT-qPCR [57]. Among all the pluripotency-inducing factors, c-Myc, Klf4 and Sox2 are the common factors expressed in regenerating tissues [29,30,58]. It is of note that we did not detect expression of *oct4* in the RPE before or after injury. Interestingly, in zebrafish, *klf4* and *oct4* are expressed in the uninjured retina and transiently increase during the process of Müller glia dedifferentiation [29]. Also in zebrafish, the knockdown of morpholino against *pou5f1* (homolog to *Oct4*) impairs fin regeneration, suggesting that Oct4 might be crucial for regeneration in this organism [28].

The process of RPE dedifferentiation was evidenced by the down-regulation of RPE specification genes *mitf* and *tyr* (tyrosinase, a melanin-catalyzing enzyme) concomitantly with an up-regulation of neural retina progenitors *ascl1* (also known as *CASH-1*, *ash1*) and *chx10* (*vsx2*) (Figure 2B,C).

We also decided to analyze if the dedifferentiated RPE cells go back into the lineage of eye formation. Different factors are crucial for eye formation; the most important are the eye field transcriptional factors that are expressed in the anterior neural plate in the region specified to become the eyes. These eye field transcriptional factors include *et*, *rx1(rx)*, *six3*, *pax6*, *lhx2*, *six6 (optx2) and tll*[59-61]. The up-regulation of *rx1*, *six6*, *lhx2* and *six3* (Figure 2B) suggests that the injury was enough to induce a transient dedifferentiation of the RPE and promote these cells to go back to the presumptive optic vesicle stage.

Despite the partial dedifferentiation of the RPE cells, by 72 h in the absence of FGF2 the RPE again acquired its pigmentation (not shown) and *mitf* expression was recovered at higher levels compared with the uninjured eye (*P* = 0.016, *n* = 3) (Figure 2C). Similar to what has been observed in Müller glia transdifferentiation in zebrafish, we observed significant up-regulation of *ascl1* (*CASH-1*), a proneural basic helix-loop-helix transcriptional factor. Importantly, *ascl1a*, the homolog to chicken *ascl1*, has been used to reprogram fibroblasts to neurons [21,62]. We also observed significant up-regulation of *rx1*, which is related to eye field specification [61,63] (Figure 2B). The importance of Rx has been demonstrated during retina regeneration in pre-metamorphic *Xenopus laevis*[64]*.*

**Figure 3:**
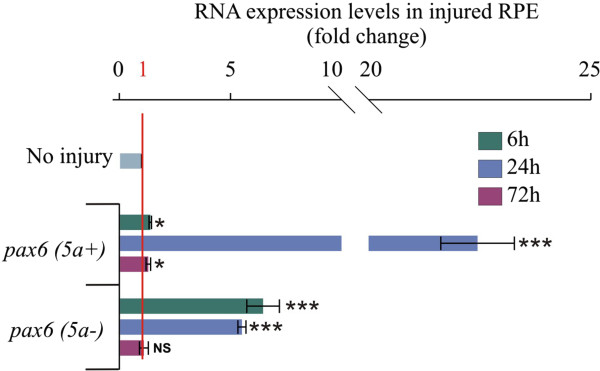
**The alternative splice variants of *pax-6 *are differentially regulated in the injured retinal pigmented epithelium.** Quantitative RT-PCR analysis of the splice variants of *pax6* (*5a-* and *5a+*) at 6, 24 and 72 h post-retinectomy (injury). The expression levels were normalized with intact retinal pigmented epithelium (no injury). The analysis was performed using three independent biological samples (*n* = 3) in triplicate and the comparative cycle threshold (2^-ΔΔCt^) method was used to determine relative changes in transcripts compared with *gapdh* mRNA levels. Significance was determined with unpaired Student’s t-test by comparing each time point with the non-injured retinal pigmented epithelium. Error bars represent standard error of the mean. *P < 0.05; ***P < 0.001, compared with intact RPE. NS, non-significant; RPE, retinal pigmented epithelium.

We next evaluated the expression of *pax6* transcriptional factor, known to be a master regulator of eye development [65-67]. Different alternative splicing variants of *pax6* have been identified in different vertebrates, with *pax6 (5a+)* and *pax6 (5a-)* being the most evolutionary conserved [68-70]. The alternative splicing of *pax6* transcript generates both forms with the variant *5a +* that has an additional 14 amino acid residues inserted in the paired domain, resulting in different specific target genes [71]. In the chick, *pax6* is expressed in retinal progenitor cells in early stages of eye development and later in ganglion, horizontal and amacrine cells [72]. To determine whether the expression of both alternative splice variants can be regulated in the injured RPE, we performed RT-qPCR using specific primers for both *pax6 (5a+)* and *pax6 (5a-)*. Although both variants were up-regulated at 6 h PR, we observed a more prominent up-regulation of *pax6 (5a-)* at 6 h PR (6.46 ± 0.73 fold-change versus 1.40 ± 0.02 for *pax6 (5a+*)). By contrast, *pax6 (5a+)* showed a higher expression at 24 h PR (22.45 ± 0.82 versus 5.5 ± 0.18 fold-change for *pax6 (5a-)*) (Figure 3). These data suggest that *pax6 (5a+)* and *(5a-)* are differentially regulated in the RPE after removing the retina. Interestingly, in the chick, when the optic vesicle is formed, the two splice variants of *pax6* are expressed in both the central nervous system and the eye primordium, with the *pax6 (5a-)* variant being the most abundant [7]. In *Xenopus laevis*, *pax6* is up-regulated in RPE cells soon after removal from the choroid, and this expression is not dependent on FGF2, although the regulation of specific variants has not been explored. In the same study, it was suggested that *pax6* expression was triggered by the alteration of the cell-extracellular matrix and/or cell-cell interactions [73,74]. It is possible that a similar effect can occur in the injured RPE in our system. Interestingly, zebrafish has two paralogs of *pax6* (p*ax6a* and *pax6b*) that are required at different points of neuronal progenitor proliferation after light-damage to the retina [75].

During mouse brain development, Pax6 (5a+) affects cell proliferation but not neural differentiation. By contrast, the canonical Pax6 (5a-) affects cell proliferation and differentiation [76,77]. It is possible that during the process of RPE transdifferentiation the alternative splice variants of *pax6* observed here have different functions or different gene targets. Collectively, these results suggest that upon retina removal at E4 (Stage 24), the quiescent cells of the RPE dedifferentiate to become progenitor-like cells similar to the cells of the optic vesicle that express the combination of eye field transcriptional factors and the factors *sox2*, *c-myc* and *klf4*. This process is transient and not sustained if no growth factors are present.

### FGF2 allows for the sustained transcriptional activity of *sox2*, *c-myc* and *klf4* in retinal pigmented epithelium undergoing reprogramming towards retina progenitors

**Figure 4:**
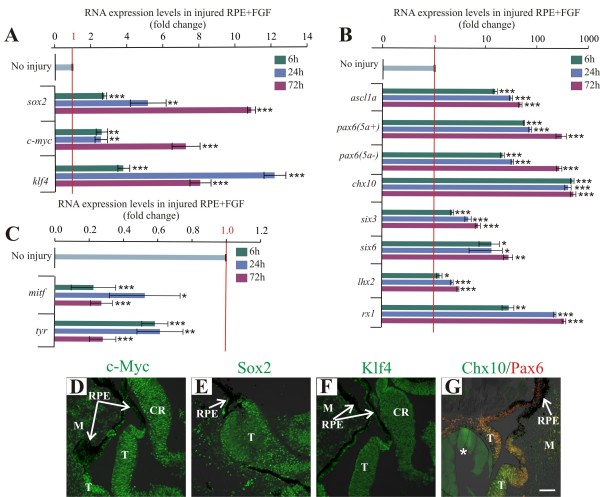
**Pluripotency-inducing factors *sox2*, *c-myc *and *klf4 *and eye field transcriptional factor expression is sustained in the injured retinal pigmented epithelium in the presence of FGF2. (A-C)** Quantitative RT-PCR analysis of *sox2*, *c-myc* and *klf4***(A)**; eye field transcriptional factors *pax6 (5a+)*, *pax6 (5a-)*, *six3*, *six6* and *lhx2*, *rx1* and the progenitor markers *ascl1*and *chx10***(B)**; and retinal pigmented epithelium (RPE)-specific markers *mitf* and *tyr***(C)** at 6, 24 and 72 h PR in the presence of FGF2. The expression levels were normalized with intact RPE (no injury). The analysis was performed using three independent biological samples (*n* = 3) in triplicate and the comparative cycle threshold (2^-ΔΔCt^) method was used to determine relative changes in transcripts compared with *gapdh* mRNA levels. Significance was determined with unpaired Student’s t-test by comparing each time point with the intact RPE (no injury). Error bars represent standard error of the mean. *P < 0.05; **P < 0.01; ***P < 0.001, compared with intact RPE. **(D-G)** Immunofluorescence analysis of c-Myc **(D)**, Sox2 **(E)**, Klf4 **(F)** and progenitor markers Pax6 and Chx10 **(G)** in transdifferentiated RPE. The asterisk represents the FGF2-soaked heparin bead. The scale bar in panel **G** represents 50 μm and applies to panels **D**-**G**. CR, ciliary regeneration; M, mesenchyme; RPE: retinal pigmented epithelium; T, transdifferentiated RPE.

To analyze the effect on the levels of expression of the pluripotency-inducing factors during the process of RPE transdifferentiation, we performed surgeries at E4 in which FGF2 heparin-coated beads were placed in the optic cup as previously described [5,6]. Thereafter, the embryos were collected at different times (6, 24 and 72 h PR) and processed for laser capture microdissection. In an attempt to avoid variation in the RPE collection, all samples were collected close to the FGF2 bead (see Methods). The RT-qPCR analysis demonstrated that the expression of *sox2* and *c-myc* was enhanced and sustained up to 72 h, when the RPE is reprogrammed towards retinal progenitors (Figure 4A). We did not observe expression of *oct4* and *nanog* under these conditions. We also evaluated the levels of expression of eye field transcriptional factors and the expression of genes associated with the RPE phenotype (*mitf* and *tyr*). Our RT-qPCR experiments demonstrated that the expression of *rx1*, *six6*, *lhx2* and *six3* was sustained at high levels (Figure 4B). In contrast with the expression patterns during injury in the absence of FGF2, the alternative splice variants *pax6 5a +* and *5a-* were up-regulated simultaneously from 6 h to 72 h (Figure 4B). RPE transdifferentiation was confirmed by a significant decrease in expression of *mitf* and *tyr* at 72 h PR (Figure 4C). Immunostaining revealed that c-Myc, Sox2, Klf4 and the retina progenitor markers Pax6 and Chx10 were present at 72 h PR in the transdifferentiated RPE (Figure 4D-G). In the absence of FGF2 at 72 h PR, the RPE did not transdifferentiate and remained pigmented, expressing only c-Myc, Klf4 and Lin-28, just as it does during normal development (Additional file 1: Figure S1F-J). As expected, in addition to transdifferentiated RPE, we also observed retina regeneration from the pool of stem/progenitor cells located in the ciliary margin of the chick eye (Figure 4D,F) [5]. Thus, we conclude that FGF2 allows the sustained expression of *sox2* and *c-myc* in retina progenitor cells along with eye field transcriptional factors to complete the transdifferentiation program of the RPE.

In agreement with our results, destruction of photoreceptors by acute light lesions in zebrafish central retina results in Müller glia dedifferentiation 4 to 8 h post-lesion, exemplified by a strong Rx1 immunoreactivity [78]. We found a significant up-regulation of *rx1* transcript at 6 h post-injury (h PR) during the transient dedifferentiation of the RPE (Figure 2B). Moreover, at 24 h post-lesion, a percentage of the activated zebrafish Müller glia cells re-enter the cell cycle, just as the chick RPE does in the presence of FGF2 (Additional file 2: Figure S2G,H). Interestingly, Müller glia cells respond by dedifferentiating after local loss of contact with photoreceptors; however, this dedifferentiation is not enough to promote re-entry to the cell cycle. Similarly, it is possible that the loss of contact between the RPE and the retina (after retinectomy) triggers the process of RPE dedifferentiation, or that the process involves an inflammation molecule such as TNFα, as in zebrafish [79], or even anaphylatoxins such as C3a or interleukins like IL-6, which have been shown to play a role in chick retina regeneration [80]. Taken together, these data suggest that similar mechanism of dedifferentiation and proliferation could be shared between retina regeneration from the RPE and from Müller glia cells.

### Overexpression of Lin-28 induces retinal pigmented epithelium transdifferentiation

**Figure 5:**
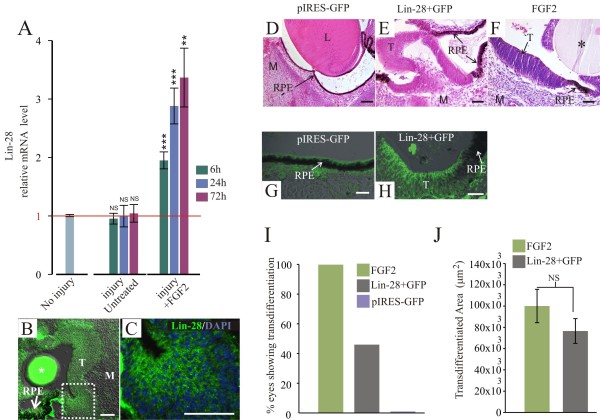
**Lin-28 is sufficient to induce retinal pigmented epithelium transdifferentiation. (A)** Quantitative RT-PCR analysis at 6, 24 and 72 h post-retinectomy (PR) shows the relative levels of lin-28 expression in the injured retinal pigmented epithelium (RPE) in the absence or presence of FGF2. The expression levels were normalized with intact RPE (no injury). The analysis was performed using three independent biological samples (*n* = 3) in triplicate and the comparative cycle threshold (2^-ΔΔCt^) method was used to determine relative changes in transcripts compared with *gapdh* mRNA levels. The Student’s t-test was used to determine significance. Error bars represent standard error of the mean. **P < 0.01; ***P < 0.001, compared with intact RPE. **(B)** Lin-28 immunofluorescence in the transdifferentiated RPE 72 h PR in presence of FGF2. **(C)** Magnification of the boxed area in B stained for Lin-28 (green) and DAPI (blue). **(D-F)** Hematoxylin-and-eosin-stained sections at 72 h PR of electroporated eyes with pcDNA3.1 + pIRES-GFP **(D)**, pCLIN-28 + pIRES-GFP (Lin-28 + GFP) **(E)** or treated with FGF2 **(F)**. **(G,H)** GFP immunofluorescence analysis of electroporated eyes with pIRES-GFP **(G)** or pCLIN-28 + pIRES-GFP (Lin-28 + GFP) **(H)**. **(I)** Percentage of eyes showing transdifferentiation at 72 h PR in the presence of FGF2 (*n* = 12, 100%); electroporated with pCLIN-28 + pIRES-GFP (Lin-28a + GFP) (*n* = 33, 48%, including the thickened depigmented RPE to full RPE transdifferentiation); or pIRES-GFP (*n* = 17, 0%). **(J)** Quantitative analysis of transdifferentiated areas observed in histological sections from eyes treated with FGF2 or electroporated with pCLIN-28 + pIRES-GFP. Error bars represent standard error of the mean. The asterisk represents the FGF2-soaked heparin bead. The scale bar in panels **B**, **E** and **F** represents 50 μm. The scale bars in panels **C**, **D**, and **G** and **H** represent 300 μm, 100 μm, and 20 μm respectively. L: lens; M: mesenchyme; NS: non-significant; RPE: retinal pigmented epithelium; T: transdifferentiated RPE.

Previously, it has been shown that Lin-28 is present in the neural tube of mouse embryos, co-localizing with Sox2. Interestingly, constitutive expression of Lin-28 in mouse embryonic carcinoma cells increases neural differentiation [33]. During human stem cell differentiation to neural progenitors, overexpression of Lin-28 rescues the proliferation deficit associated with absence of Sox2, suggesting that Lin-28 is important for proliferation of neural progenitors cells [81]. In zebrafish, upon retinal injury, Müller glia cells express the proneural gene *ascl1a* along with *lin-28,* generating a regulatory loop in which *ascl1a* regulates *lin-28*, which in turn negatively regulates the miRNA *Let-7*[29]. Because both *sox2* and the *ascl1* are expressed during the process of RPE transdifferentiation (Figures 4A,B,E), we decided to investigate the regulation of *lin-28* expression in the injured eye and during FGF2-induced transdifferentiation. Interestingly, in comparison with *sox2*, *c-myc* and *klf4*, we found that *lin-28* was significantly up-regulated in the presence of FGF2, but not with injury alone (Figure 5A). Consistent with our RT-qPCR results, Lin-28 was detected in the transdifferentiated RPE at 72 h PR showing a cytoplasmic pattern (Figures 5B,C). Given that *lin-28* mRNA levels are only up-regulated in the presence of FGF2, it is possible that *lin-28* could play a role in completing the transdifferentiation process. Thus, we wondered if *lin-28* overexpression could be sufficient to induce RPE transdifferentiation in the absence of FGF2. To address this question, we co-electroporated retinectomized chick eyes with a plasmid containing chicken *lin-28* (pCLin28a) and pIRES-GFP (to monitor the electroporated areas, see immunostaining in Figure 5H) or co-electroporation of pcDNA3.1 and pIRES-GFP as controls (Figure 5G), and the embryos were collected 72 h PR. The systematic analysis of histological sections showed a range of effects on the RPE varying from clear thickened depigmented areas to full transdifferentiation (*n* = 33, 48%, Figure 5I). This range of effects is most likely due to the electroporation efficiency. Unlike the remarkable effects observed with *lin-28* overexpression, pIRES-GFP (control plasmid) electroporation did not show evidence of transdifferentiation or lack of pigmentation (Figure 5D,G). Furthermore, overexpression of Lin-28 recapitulated FGF-induced transdifferentiation (compare Figure 5E and F) as well as the amount of transdifferentiated area (Figure 5J, *P* = 0.2356 comparing Lin-28 and FGF2). Collectively, these results demonstrate that Lin-28 is sufficient to induce RPE transdifferentiation in the absence of an external source of FGF2.

## Conclusion

**Figure 6:**
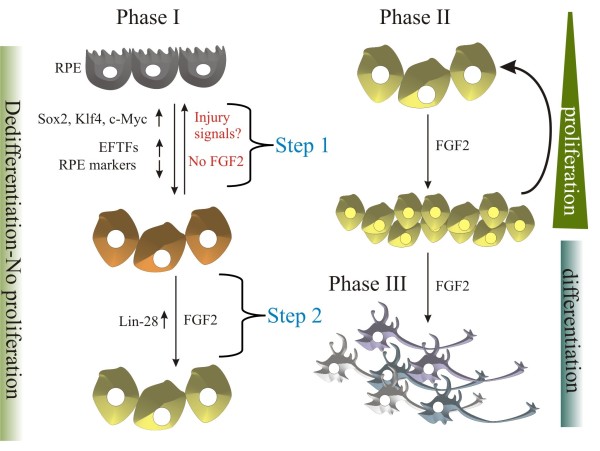
**Model representing the process of chick retinal pigmented epithelium transdifferentiation.** Phase I includes dedifferentiation. During this phase, there is no proliferation. During step 1, injury signals are produced in response to retinectomy; pluripotency-induced factors Sox2, c-Myc and Klf4 that are also present in retina progenitors are up-regulated as well as eye field transcriptional factors (EFTFs), along with a down-regulation of RPE specific markers. During step 2, after the addition of exogenous FGF2, Lin-28, is up-regulated; however, during this stage there is no cell proliferation. In Phase II, in the presence of FGF2, proliferation is initiated. Lastly, on Phase III, differentiation of retinal cells takes place.

We have identified a series of factors that are up-regulated with injury only (step 1) including the factors *sox2*, *c-myc* and *klf4* and eye field transcriptional factors. In addition, we have found that *lin-28*, a pluripotency-inducing factor and a microRNA regulator that is also a critical player in other systems of regeneration, is only up-regulated upon addition of FGF2 (step 2) in the chick RPE after retina removal, at a time where there is no cell proliferation (Figure 6). Finally, we demonstrated that Lin-28 is sufficient to induce RPE transdifferentiation in chick retinectomized eyes in the absence of exogenous FGF2. The conservation of a dedifferentiation molecular profile between regenerative models including retina, lens and limb or fin regeneration is indicative of a common process to reprogram cells to a plastic state, where the cells can be directed to expand and respond to environmental cues in order to differentiate and replace lost cells and tissues.

## Methods

### Chick embryos and surgical procedures

Fertilized Specific Pathogen-Free chicken eggs (Charles River Laboratories, Wilmington, MA, USA) were incubated in a humidified rotating incubator at 38°C. At E4 (Stage 24 [4]), retinectomies and FGF2 treatments were performed as previously described [5]. Embryos were collected at 6, 24 and 72 h PR and processed for laser capture microdissection, histology and immunofluorescence. For proliferation studies, 10 μl of BrdU (10 μg/ml) solution was dropped over the eye of the embryo 1 h before collection.

### Laser capture microdissection

Laser capture microdissection was performed as previously described [80]. Briefly, embryos were collected and infiltrated at 4°C with 6.25%, 12.5% and 25% sucrose for 10, 20 or 30 min, respectively, followed by 2:1 25% sucrose to optimal cutting temperature (OCT) compound (Sakura Finetek, Torrance, CA, USA) for 1 h and frozen in dry ice and methylbutane. Cryosections (12 μm) were collected onto metal-framed polyethylene naphthalate membrane slides (Arcturus, Applied Biosystems, Foster City, CA, USA), fixed in 70% ethanol at -20°C for 30 s, rinsed in cold diethylpyrocarbonate-treated water, stained with hematoxylin (Sigma, St. Louis, MO, USA) for 10 s, and dehydrated in ethanol for 30 s each in 70%, 95% and finally 2 min in 100% ethanol. Laser capture microdissection was performed using a Veritas laser capture microdissection system and software as described previously [80]. Laser microdissected sections were collected in CapSure HS LCM Caps (Molecular Devices, Foster City, CA, USA), and total RNA extraction was performed using PicoPure RNA Isolation Kit (Arcturus, Applied Biosystems) including a treatment with DNase I (RNase-Free DNase Set; Qiagen, Darmstadt, Germany). The quality and quantity of RNA were determined using an Agilent RNA 6000 Pico LabChip (Agilent 2100 Bioanalyzer; Agilent Technologies, Santa Clara, CA, USA). Five nanograms of total RNA with an RNA integrity number > 8 were amplified using Ovation Pico WTA System V2 (NuGEN, San Carlo, CA, USA) according to the manufacturer’s instructions, to generate the Single Primer Isothermal Amplification (SPIA) cDNA. Finally, SPIA cDNA was purified using QIAquick PCR Purification Kit (Qiagen) and quantified using a Nanodrop ND-100 spectrophotometer (Thermo Fisher Scientific, Kalamazoo, MI, USA).

### RT-PCR

Total RNA was extracted from Stage 8 whole embryos using NucleoSpin RNA II isolation Kit (Macherey-Nagel, Düren, Germany) following the manufacture’s protocol. The quality and quantity of RNA were determined using Agilent RNA Nano LabChip (Agilent 2100 Bioanalyzer; Agilent Technologies). Approximately 300 ng of total RNA with a an RNA integrity number > 8 were used for cDNA synthesis using ImProm-II Reverse Transcription System (Promega, Madison, WI, USA) and random-primer hexamers according to the manufacturer’s instructions. For CM and RPE, the amplified SPIA cDNA (50 ng) was used as a template in the PCR reactions. All PCR reactions were performed using PlatinumTaq DNA polymerase (Invitrogen, Grand Island, NY, USA) and the intron-spanning primers are listed in Additional file 3: Table S1. Amplification conditions included denaturation at 95°C for 1 min, annealing at 55°C for 2 min, and extension at 72°C for 30 s. All the RT-PCR reactions were run from at least three independent biological samples and the fragments were gel-purified and sequenced to confirm the specificity of the sequence.

### Quantitative RT-PCR

RT-qPCR was performed using a 20 μl mixture containing 5 μl (4 ng) SPIA cDNA, 10 μl 2× SYBR Green/Fluorescein qPCR Master Mix (SABiosciences, Qiagen, MD, USA), and a 500 nM final concentration of the primers. Splice junction-specific primers were designed using Primer 3 (v 4.0) available in [82] and were optimized following guidelines for RT-qPCR experiments (amplification efficiency and melting curves) [83]. Primers sequences and Ensembl or GenBank identification numbers are provided in Additional file 3: Table S2. Amplifications reactions were performed in triplicate using an iCycler (BioRad, Hercules, CA, USA). The cycling conditions included 10 min polymerase activation at 95°C and 35 cycles of 15 s at 95°C and 1 min at 60°C, followed by a dissociation run from 65°C to 95°C for melting curve analysis. The comparative cycle at threshold (2^-ΔΔCt^) and an unpaired Student’s t-test analysis were used to determine relative changes in transcript levels compared to *gapdh* mRNA levels as previously reported [84] using SigmaPlot 8.0 Software. All analyses were performed in triplicate with at least three independent biological samples.

### Antibodies

Antibodies against Sox-2 (1:100), c-Myc (1:50) and Lin-28 (1:100) were purchased from Santa Cruz Biotechnology. Antibody against Klf4 (1:100) was purchased from Aviva Systems Biology (San Diego, CA, USA). Antibodies against Mitf (1:500), GFP (1:50) and BrdU (1:50) were purchased from Abcam (Cambridge, MA, USA). Antibody against Pax-6 (1:10) was obtained from the Developmental Studies Hybridoma Bank (Iowa City, IA, USA). Anti-Chx-10 (1:250) antibody was purchased from ExAlpha (Shirley, MA, USA). Antibody against p27^Kip1^ (1:50) was obtained from BD Biosciences (Franklin Lakes, NJ, USA). All secondary antibodies were purchased from Molecular Probes (Grand Island, NY, USA) and used at 1:100 dilution.

### Immunohistochemistry

Embryos were fixed in 4% paraformaldehyde in PBS for 4 h at room temperature, equilibrated in 30% sucrose, embedded in OCT compound (Sakura Finetek), and sectioned at 12 μm. For the p27^Kip1^ antibody, tissues were fixed in 10% neutral buffered formalin (Thermo Scientific), embedded in paraffin, sectioned, and deparaffinized followed by 30 min antigen retrieval. Sections were permeabilized with 1% saponin in PBS or 15 min in 2 N HCl in PBS for BrdU immunostaining and blocked with 10% goat or donkey serum and incubated overnight at 4°C with the primary antibody. Sections were incubated in secondary antibody and coverslipped with Vectashield (Vector Laboratories, Burlingame, CA, USA). Confocal images (size 1,024 × 1,024) were collected sequentially on a Zeiss 710 Laser Scanning Confocal System (Jena, Germany) using a × 20/0.80 Numeric Aperture (NA) = 0.55 WD objective lens or EC Plan-Neofluar. Results were confirmed using three different biological samples.

### Lin-28a construct and in ovo electroporation

Chicken Lin-28a was cloned from a Stage 14 embryo as previously described [42] and the cDNA was synthetized as described in the RT-PCR section using the primers described in Additional file 3: Table S3. The PCR product was gel-purified and cloned in pDrive plasmid to generate pDlin28a plasmid (Qiagen). Thereafter, a *Hind*III/*BamH*I fragment of 895 base pairs from pDlin28a was cloned using the same restriction sites in pcDNA3.1+ (Invitrogen) to generate pCLin28a. T7 primer was used to confirm the integrity of the Lin-28a sequence. Electroporations were performed 1 h PR by injecting 3 μl of a mixture of pCLin28a and pIRES-GFP (1:1, 3 μg each) or 3 μl of a mixture of pcDNA3.1 and pIRES-GFP as controls at the same concentration. The injections were performed using a Pico-injector system PLI-100 (Harvard Apparatus, Holliston, MA, USA) and glass capillary needles. Thereafter, a gold-plated wire electrode (In Ovo gene model 512; BTX Technologies inc., Bent, Holliston, MA, USA), used as an anode, was placed in the ventral border of the eye and a platinum and iridium electrode (FHC Inc., Bowdoinham, ME, USA), used as cathode, was inserted on the top of the brain. Three square pulses of 15 V of 50 ms length and at 950 ms intervals were applied using an ECM 830 electroporator (BTX Technologies, Inc.). The window on the shell was sealed and the embryos were returned to the incubator and collected 72 h post-electroporation and processed for immunohistochemistry and histology.

### Histology and quantification

Embryos used for histological analysis were fixed in Bouin’s fixative (Ricca Chemical Company, Arlington, TX, USA), embedded in paraffin, sectioned at 12 μm, stained with hematoxylin and eosin, and photographed using an Olympus BX-51 microscope (Tokio, Japan). To quantify the amount of transdifferentiated RPE, we analyzed the area from three histological sections of four different eyes (12 sections). Images were captured using an Olympus camera and Magnafire image capture software and processed using ImageJ software available in [85]. The transdifferentiated area was calculated using the free hand tool and a two-tailed permutation test for comparing means using R version 3.0.

## Abbreviations

BrdU: 5-bromo-2′-deoxyuridine; E: embryonic day; FGF: fibroblast growth factor; h PR: hours post-retinectomy; miRNA: microRNA; OCT: optimal cutting temperature; PBS: phosphate-buffered saline; PCR: polymerase chain reaction; qPCR: quantitative polymerase chain reaction; RT: reverse transcriptase; SPIA: single primer isothermal amplification; TNFα: tumor necrosis factor alpha.

## Competing interests

The authors declare that they have no competing interests.

## Authors’ contributions

AL-M designed and performed experiments, analyzed data and co-wrote the paper. EG-E, AM, AMD and KB-S performed experiments and analyzed data. PAT discussed experiments, analyzed data and co-wrote the paper. KDRT conceived the idea, supervised the work, designed experiments, analyzed the data and co-wrote the paper. All authors have read and approved the final manuscript.

## Supplementary Material

Additional file 1: Figure S1 Pluripotency inducing factors are present in the developing chick eye at E7 and after injury. **(A-E)** Immunohistochemical staining using antibodies against pluripotency inducing factors c-Myc **(A)**, Sox2 **(B)**, Klf4 **(C)**, Lin‒28 **(D)** and progenitor markers Pax6 and Chx10 **(E)** in the posterior region of E7 (this stage was included for comparison with the 72 h PR, as the developing retina would be equivalent to the regenerating retina 72 h PR) chick eyes. Higher magnification view of the boxed areas are shown in A’-E’; dashed lines outline the RPE. **(F-J)** Immunofluorescence analysis of c‒Myc **(F)**, Sox2 **(G)**, Klf4 **(H)**, Lin-28 **(I)** and progenitor markers Pax6 and Chx10 **(J)** in eyes 72 h PR. **(K-M)** Negative controls (only secondary antibodies added) for immunofluorescence analysis on eyes 72 h PR for Sox2 and Lin-28 **(K)**, c‒Myc and Klf4 **(L)** and Pax6 and Chx10 **(M)**. L, lens; M, mesenchyme; **R**, retina; RPE, retinal pigmented epithelium; Asterisk, FGF2-soaked heparin bead. The scale bar in panel E represents 50 μm and applies to panels **A-E**. Scale bar in panel **E’** represents 10 μm and applies to panels A’-E’. The scale bar in panel **J** represents 50 μm and applies to panels **F-J**. The scale bar in panel **M** represents 50 μm and applies to panels **K-M**. Click here for file

Additional file 2: Figure S2 FGF2 induces proliferation of the RPE. **(A-H)** Retinectomies were performed on E4 (Stage 24) chick eyes in the absence **(A-D)** or presence **(E-H)** of FGF2. Immunostaining using an antibody to detect BrdU **(A,C,E,G)**, shows BrdU + RPE cells only at 24 h post-retinectomy (PR) in the presence of FGF2. Immunostaining to detect p27kip1 **(B,D,F,H)** is negative in the RPE only at 24 h PR in the presence of FGF2. L, lens; M, mesenchyme; RPE, retinal pigmented epithelium; *Asterisk, FGF soaked heparin bead. The scale bar in panel H represents 50 μm and applies to all images. Click here for file

Additional file 3: Table S1 Primer sequences for RT-PCR. **Table S2.** Primer sequences for RT**-**qPCR. **Table S3.** Primer sequences for *lin*-*28* cloning. NCBI accession number NM_001031774. Click here for file
