# Supplementary figures and images for: Reprogramming of the chick retinal pigmented epithelium after retinal injury
Source: BMC Biol. 2014 Apr 17;12:28. doi: 10.1186/1741-7007-12-28 (PMC4026860; doi:10.1186/1741-7007-12-28)

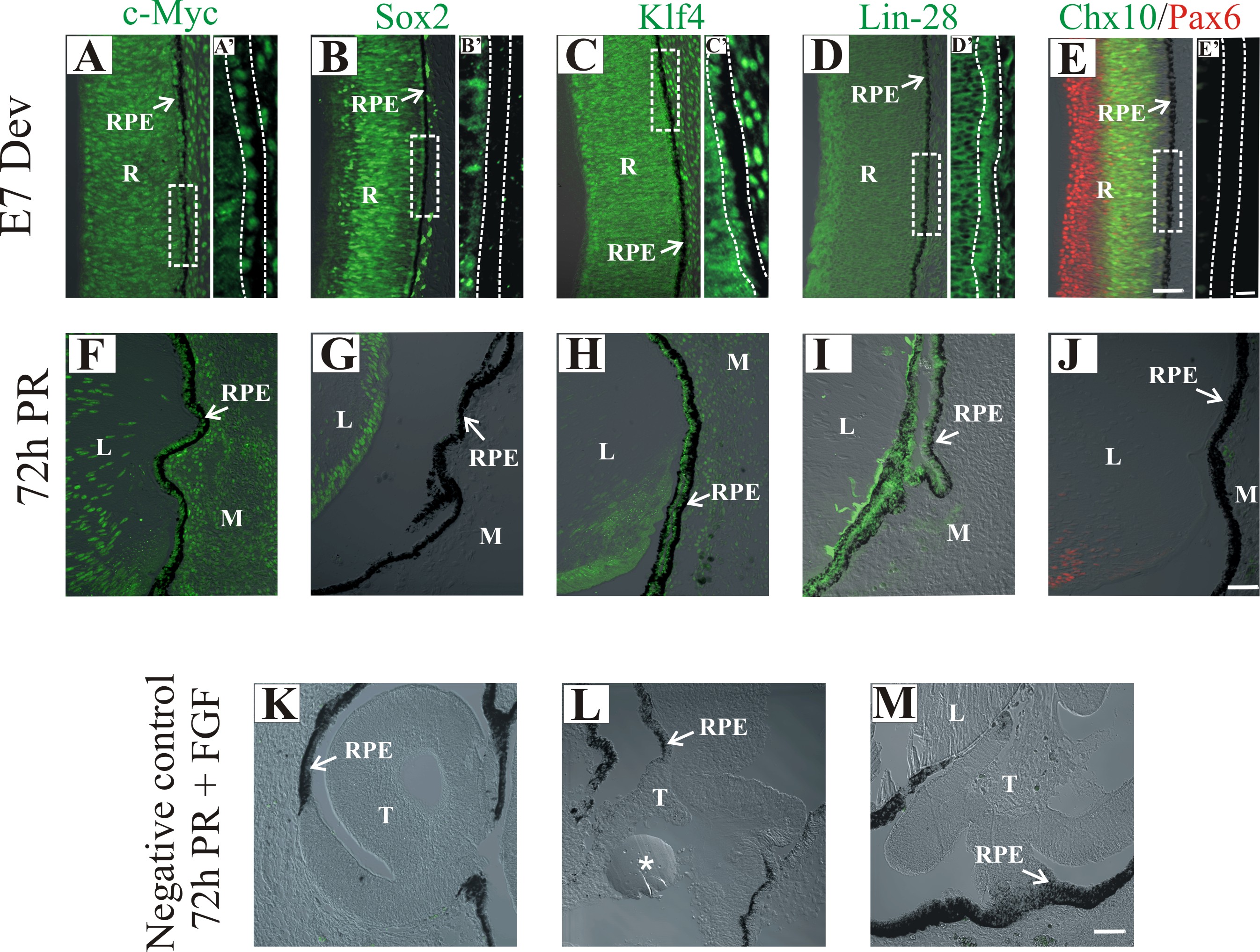

Supplement: Additional file 1: Figure S1 — Pluripotency inducing factors are present in the developing chick eye at E7 and after injury. (A-E) Immunohistochemical staining using antibodies against pluripotency inducing factors c-Myc (A), Sox2 (B), Klf4 (C), Lin‒28 (D) and progenitor markers Pax6 and Chx10 (E) in the posterior region of E7 (this stage was included for comparison with the 72 h PR, as the developing retina would be equivalent to the regenerating retina 72 h PR) chick eyes. Higher magnification view of the boxed areas are shown in A’-E’; dashed lines outline the RPE. (F-J) Immunofluorescence analysis of c‒Myc (F), Sox2 (G), Klf4 (H), Lin-28 (I) and progenitor markers Pax6 and Chx10 (J) in eyes 72 h PR. (K-M) Negative controls (only secondary antibodies added) for immunofluorescence analysis on eyes 72 h PR for Sox2 and Lin-28 (K), c‒Myc and Klf4 (L) and Pax6 and Chx10 (M). L, lens; M, mesenchyme; R, retina; RPE, retinal pigmented epithelium; Asterisk, FGF2-soaked heparin bead. The scale bar in panel E represents 50 μm and applies to panels A-E. Scale bar in panel E’ represents 10 μm and applies to panels A’-E’. The scale bar in panel J represents 50 μm and applies to panels F-J. The scale bar in panel M represents 50 μm and applies to panels K-M. [file 1741-7007-12-28-S1.jpeg]

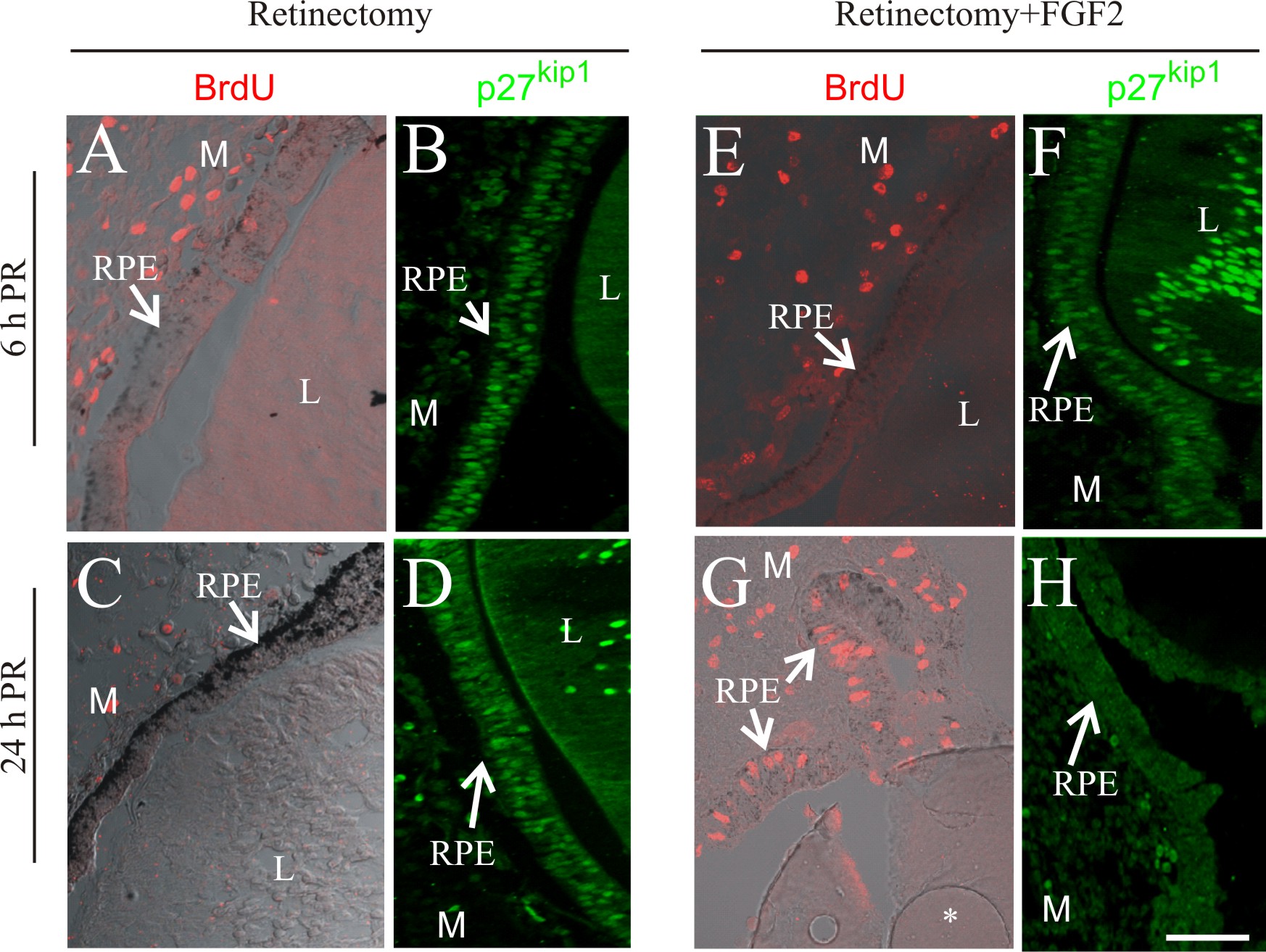

Supplement: Additional file 2: Figure S2 — FGF2 induces proliferation of the RPE. (A-H) Retinectomies were performed on E4 (Stage 24) chick eyes in the absence (A-D) or presence (E-H) of FGF2. Immunostaining using an antibody to detect BrdU (A,C,E,G), shows BrdU + RPE cells only at 24 h post-retinectomy (PR) in the presence of FGF2. Immunostaining to detect p27kip1 (B,D,F,H) is negative in the RPE only at 24 h PR in the presence of FGF2. L, lens; M, mesenchyme; RPE, retinal pigmented epithelium; *Asterisk, FGF soaked heparin bead. The scale bar in panel H represents 50 μm and applies to all images. [file 1741-7007-12-28-S2.jpeg]
