# Supplementary material for: Reprogramming of the chick retinal pigmented epithelium after retinal injury
Source: BMC Biol. 2014 Apr 17;12:28. doi: 10.1186/1741-7007-12-28 (PMC4026860; doi:10.1186/1741-7007-12-28)
Supplement: Additional file 3: Table S1 — Primer sequences for RT-PCR. Table S2. Primer sequences for RT-qPCR. Table S3. Primer sequences for lin-28 cloning. NCBI accession number NM_001031774. [file 1741-7007-12-28-S3.pdf]

Table S1. Primer Sequences for RT-PCR

Gene	Ensembl or GenBank ID	Sequence 5'-3'
oct4 (*cPouV*)	ENSGALT00000043197	F, CAATGCAGAGCAAGTGCTGG
		R, TCTGGAGCTGAAGCTGTTTG
nanog	ENSGALT00000044516	F, TTTCAGAGCCAGAAGTACCTC
		R, GTCATATCCAGATACGCAGC
lin-28a	ENSGALT00000000529	F, GGGTCTGTTTCCAACCAGCAGT
		R, ATCGACGGGTGAGTCCAGCAT
sox2	ENSGALT00000014379	F, ACCCTTCATTGACGAAGCC
		R, TAACTGTCCATCCTCTGGTTC
klf4	ENSGALT00000042652	F, AAGAACAGCCACTCACACCTG
		R, AAATGCTCTGTCACACCGCTG
c-myc	ENSGALT00000026309	F, CCACGACCAGCAGCGACT
		R, CGCAGGGCAAAGAAACTCAG
rdh10	ENSGALG00000015640	F, AGGAATGTTCAGAGGGTGCAGGAT
		R, ATACATGAGGCGAGGTGTGCAGAT
mitf	ENSGALT00000012448	F, AAAGCATGCCTCCTCCAGGACTTA
		R, TTGGGTATCAAGGTGCCCAGTTCT
gapdh	ENSGALG00000014442	F, TCCAAACTCATTGTCATACCAGGAA
		R, ACCACTGTCCATGCCATCACAGCC

Table S2. Primer Sequences for RT-qPCR

Gene	Ensembl or GenBank ID	Sequence 5'-3'
sox2	ENSGALT00000014379	F, TGAACGGATCGCCTACCTAC
		R, CTGGATTCCGTCTTGACCAC
c-myc	ENSGALT00000026309	F, CAGCAGCGACTCGGAAG
		R, CTTCTGTGCTGGACTCTGTG
klf4	ENSGALT00000042652	F, ACCAAGAGCTCTCATCTCAAGGCA
		R, ATCAGATCGGGCAAACTTCCATCC
mitf	ENSGALT00000012448	F, ACATCAGCAACTCCTGTCCA
		R, TTGCCTCTCCTTAGCCAGTG
tyr	ENSGALT00000027865	F, TTTGCTGATCCACACACTGC
		R, GATCATTCGCAGAGCCTTGT
ascl1 (*CASH-1*, *ash1*)*	NM_204412.1	F, TCAACTTCAGCGGCTTCG
		R, AGGTTCACCAGCTTGACTCG
chx10 (*vsx2*)*	ENSGALT00000016606	F, GACCGCAAAATGTCCAAATC
		R, GCCTTTTCCAGTTCTTCCAG
six3	NM_204364.1	F, CCCCACGAAGAGTTGTCAAT
		R, TATGTCTCCGGTCTCCTCCA
six6 (*optx2*)*	ENSGALT00000037367	F, CCACACGGGGAACTACAGAG
		R, GCTTCCAGCCAGAGAGCTT
lhx2	ENSGALT00000001693	F, CTGCAATGAAAACGATGGTG
		R, GGTGGTGTTTGAATGATGTCC
rx1	ENSGALT00000032299	F, CCCACTACCCCGACGTGTA
		R, CTGGCCTCCATCTTCTCCTG

Table S2. Primer Sequences for RT-qPCR

Gene	Ensembl or GenBank ID	Sequence 5'-3'
pax6 5a+	ENSGALT00000019804	F, GGCAGAAGATCGTGGAACTC
		R, CAGCACTTGGACTTTTGCAT
pax6 5a-	ENSGALT00000019805	F, GGCAGAAGATCGTGGAACTC
		R, TTCGTAATACCTGCCCAAAA
lin-28a	ENSGALT00000000529	F, ACTCACCCGTCGATGTCTTC
		R, TGGAGGATTTCTTGAAGGTGA

*Alternative names in parenthesis.

Table S3. Primer Sequences for *lin-28* cloning (NCBI Accession number NM_001031774)

Sequence 5'-3'
F, GGATGGGGTCTGTTTC
R, TGCCATCATTCCCGGGTT
